# The chronification mechanism of orofacial inflammatory pain: Facilitation by GPER1 and microglia in the rostral ventral medulla

**DOI:** 10.3389/fnmol.2022.1078309

**Published:** 2023-01-06

**Authors:** Wenwen Zheng, Xilu Huang, Jing Wang, Feng Gao, Zhaowu Chai, Jie Zeng, Sisi Li, Cong Yu

**Affiliations:** ^1^The Affiliated Hospital of Stomatology, Chongqing Medical University, Chongqing, China; ^2^The Sixth People’s Hospital of Chongqing, Anesthesiology, Chongqing, China

**Keywords:** chronic orofacial pain, GPER1, microglia, GluA1, neuroplasticity

## Abstract

**Background:**

Chronic orofacial pain is a common and incompletely defined clinical condition. The role of G protein-coupled estrogen receptor 1 (GPER1) as a new estrogen receptor in trunk and visceral pain regulation is well known. Here, we researched the role of GPER1 in the rostral ventral medulla (RVM) during chronic orofacial pain.

**Methods and Results:**

A pain model was established where rats were injected in the temporomandibular joint with complete Freund’s adjuvant (CFA) to simulate chronic orofacial pain. Following this a behavioral test was performed to establish pain threshold and results showed that the rats injected with CFA had abnormal pain in the orofacial regions. Additional Immunostaining and blot analysis indicated that microglia were activated in the RVM and GPER1 and c-Fos were significantly upregulated in the rats. Conversely, when the rats were injected with G15 (a GPER1 inhibitor) the abnormal pain the CFA rats were experiencing was alleviated and microglia activation was prevented. In addition, we found that G15 downregulated the expression of phospholipase C (PLC) and protein kinase C (PKC), inhibited the expression of GluA1, restores aberrant synaptic plasticity and reduces the overexpression of the synapse-associated proteins PSD-95 and syb-2 in the RVM of CFA rats.

**Conclusion:**

The findings indicate that GPER1 mediates chronic orofacial pain through modulation of the PLC-PKC signal pathway, sensitization of the RVM region and enhancement of neural plasticity. These results of this study therefore suggest that GPER1 may serve as a potential therapeutic target for chronic orofacial pain.

## 1. Introduction

Chronic pain has become a major cause of economic burden around the world (Jackson et al., 2015). About 7%–11% of people suffer from chronic orofacial pain. Correct diagnosis and management of the condition can be difficult due to the complexity of the orofacial anatomy, limited knowledge of its pathophysiology, and limited treatment options (Handa et al., 2021). This is further complicated by the lack of clarity on the disease’s comorbid features and its pathogenesis (Slade et al., 2020). The orofacial regions are vital to sustaining life with multiple functions (Iwata and Sessle, 2019) and the orofacial sensorimotor system is unique and distinct from the spinal sensorimotor system (Avivi-Arber and Sessle, 2018). Therefore, in order to solve the problems caused by chronic orofacial pain, it is necessary to study its possible central mechanism and find new therapeutic targets.

The rostral ventral medulla (RVM), consisting of the large nucleus of the middle suture, the giant reticular nucleus, and the giant cell nucleus, is considered a key nucleus involved in both down-regulation inhibition and down-regulation facilitation pathways. It does so by projecting directly to the spinal cord dorsal horn (SCDH) and medullary dorsal horn (MDH; Ossipov et al., 2000; Burgess et al., 2002). Early neuroanatomical studies have shown that RVM could modulate injurious inputs because its downward projection could form synaptic connections with primary afferent terminals, projection neurons, and interneurons (Chen and Heinricher, 2019; Bagley and Ingram, 2020).

The G protein-coupled estrogen receptor 1 (GPER1) contains seven transmembrane structural domains, and is rapidly activated by an extracellular signal-regulated kinase (ERK) that increases aberrant pain. It is a plasma membrane protein widely distributed in different organs (Brailoiu et al., 2007; Olde and Leeb-Lundberg, 2009; Akama et al., 2013), and can induce rapid non-genomic estrogenic signaling (Revankar et al., 2005). At present, estrogen and this receptor play an important role in chronic pathological pain, and are thought to attribute to the higher prevalence of poorly controlled chronic pain observed in females. Nevertheless GPER1 is widely distributed in males as well and plays an important role in chronic pain for both sexes (Gao et al., 2017). Similarly, studies have shown that the distribution of GPER1 in rats is independent of sex (Brailoiu et al., 2007). In the nerve injury model, microglia have been confirmed to be activated in the RVM, leading to the increase of proinflammatory factors (Wei et al., 2008; Wang et al., 2019). Neuroglia activation in the RVM and their involvement in pain modulation were confirmed in a nerve injury model (Dubovy et al., 2018). Recent studies indicate that GPER1 is present in both neurons and microglia and is involved in neuroinflammation and neuropathic pain modulation (Dubovy et al., 2018; Correa et al., 2020).

Central sensitization is considered the main mechanism underlying chronic pain. Our previous studies have shown that the main factor of chronic pain is peripheral and central sensitivity (Zeng et al., 2018; Bai et al., 2019). Neuron-glial cell interactions were proposed *via* glutamate, in which microglia play an important role (Hagino et al., 2004).Recent studies have sought to understand the central sensitizing effect of phospholipase C-protein kinase C (PLC-PKC) on chronic pain (Galeotti et al., 2006; Wei et al., 2016). Moreover, estrogen activates PKC, but it is GPER1 rather than the classical estrogen receptors (Rα and Rβ) that produce this effect (Hucho et al., 2006). Given the above evidence, we hypothesized that the development of chronic orofacial pain is influenced by the activation of the GPER1 in the microglia of the RVM, which subsequently drives the release of glutamate and produces changes in synaptic plasticity, which can serve as a complementary central mechanism based on previous studies.

In this study, we investigated the mechanism which GPER1 and microglia in the RVM influence and facilitate chronic orofacial pain. By establishing a chronic pain model in rats, evaluating their pain thresholds and histopathological analysis of the levels of GPER1 and other associated markers of chronic pain and sensitization, we were able to elucidate that GPER1 facilitates chronic orofacial pain by a variety of mechanisms and may serve as an important way to treat chronic orofacial pain.

## 2. Materials and methods

### 2.1. Animals

Sprague–Dawley adult rats (male, weighing 250–300 g) were obtained from the Experimental Animal Center of Chongqing Medical University (Chongqing, China). Table 1 shows the group. Rats were housed at 23 ± 1°C under a 12 h light/dark cycle. Let the rats adapt to the environment for at least 7 days. The Ethics Committee of the Stomatology Hospital affiliated Chongqing Medical University approved the study (registration no. CQHS-NT022-2022).

**Table 1:** Animal numbers in each group, administration time (day, *D*), and sample size (number, *n*) of rats per group.

Experimental group	D1–7	D8	WB (*n*)	IF (*n*)	Threshold
Control (CON)	NS	-	6	3	6^a^
CFA	CFA	-	6	3	6^a^
CFA + DMSO	CFA	0.1%DMSO	6	3	6^a^
CFA + U73122	CFA	U73122 (mmol)	-	-	6
CFA + GF109203x	CFA	GF109203x (mmol)	-	-	6
CFA + G15	CFA	G15 (mmol)	6	3	6^a^
CFA + G15 + PMA	CFA	G15 + PMA (μmol)	-	-	6
CFA + G15 + m-3M3FBS	CFA	G15 + m-3M3FBS (μmol)	-	-	6

a Indicates shared with other experiments, do not count.

WB: western blot; IF: immunofluorescence; NS: Physiological saline; PMA: Phorbol 12-myristate 13-acetate.

### 2.2. Catheter placement

All rats were implanted with catheters before receiving drug injections into the temporomandibular joint (TMJ) cavity to observe the effect of pharmacological inhibitors or agonists of GPER1, PLC, and PKC on nociceptive hypersensitivity due to CFA-induced temporomandibular osteoarthritis. All procedures were performed in a large pool. The operation has been described previously (Gao et al., 2017). Before continuing the experiments, the rats were allowed to recover for 1 week.

### 2.3. Drugs injection

One week later, the rats were anesthetized and administered separate intracerebral drugs *via* injection. The GPER1 inhibitor (G15, 10 μM, 5 μL, *n* = 6, MCE, Monmouth Junction, NJ, USA) or 0.1% dimethyl sulfoxide (DMSO; CFA + Vehicle group; 5 μL, *n* = 6, Mengbio, Chongqing, China) was injected through the catheter 0.5 h before behavioral testing. The PKC agonist (PMA, 65 μM, 5 μL, MCE, Monmouth Junction, NJ, USA), PKC inhibitor (GF109203x, 4 mM, 5 μL, *n* = 6, MCE, Monmouth Junction, NJ, USA), PLC agonist (m-3M3FBS, 60 μM, 5 μL, MCE, Monmouth Junction, NJ, USA), and PLC inhibitor (U73122, 5 mM, 5 μL, *n* = 6, MCE, Monmouth Junction, NJ, USA) were injected through the catheter 24 h before behavioral testing.

### 2.4. Behavioral assays

One week after intra-articular drug injection, the rats were tested with the von Frey hair test (User Manual, Dan Mic Global, LLC. Camden Avenue, San Jose, CA, USA) to detect the head withdrawal threshold (Fruhstorfer et al., 2001). The rats were placed in acrylic cages and adapted to the environment for approximately 30 min. Von Frey hair was applied on the skin above the TMJ cavities of the rats, and the force applied toward the area increased gradually. Positive responses included (1) stimulated behavior, characterized by the exhibition of rapid backward movement, curling up against the cage wall to avoid further contact with the face, or hiding the head and face from the hairs to avoid the stimulus; (2) aggressive behavior, characterized by the exhibition of rapid grasping and biting of the stimulus, resulting in aggressive movement; and (3) stroking the face with the ipsilateral front paw or making a sound. The presence of one or more of the above responses was considered positive in the stimulation test. The interval for each test was greater than 1 min. The head withdrawal threshold of the rats was measured using von Frey hair 1 h before and after the first CFA injection daily. The test was performed after each catheter infusion (24 h after GF109203x and U73122 injection and 0.5 h after G15 injection) and before injection. The details of test procedures and experimental data processing have been described (Chaplan et al., 1994; Ren, 1999; Cao et al., 2009).

### 2.5. Establishment of temporomandibular joint

Rats were randomly divided into control (CON) and CFA groups. Rats in the CFA group were anesthetized by isoflurane (same dose as used before), and 50 μL of CFA (Sigma-Aldrich, 3,050 Spruce Street, Saint Louis, USA) was injected bilaterally into the joint cavity using an insulin syringe to cause TMJ inflammation (Wu et al., 2010). One week after CFA injection, the TMJ areas showed severe swelling (Li et al., 2016). Rats in the CON group were injected bilaterally with 50 μL 0.9% saline under the same anesthesia mode. Inflammation was assessed using head withdrawal threshold and joint histopathological examinations.

### 2.6. Histopathological examination of temporomandibular joint cartilage

The TMJ cartilage was collected following sacrifice of the rats. The specimens were fixed in 4% paraformaldehyde (Monbiot, Chongqing, China), decalcified in 10% EDTA decalcifying solution about 1.5 months, dehydrated under the ethanol concentration gradient, and the cartilage was embedded in paraffin. The specimen was then cut into 5 μm sections on a paraffin sectioning machine (Leica RM2245, Wetzlar, Germany). Paraffin sections were dewaxed and joint staining was performed using a modified Safranine O-Fast Green FCF Cartilage Stain Kit (Solarbio, Beijing, China). After staining, the specimen was sealed with resin (Solarbio, Beijing, China). Images were obtained using a microscope (Slideview VS200, Olympus Corporation, Tokyo, Japan).

### 2.7. Western blot

The expression levels of the total GluA1, GPER1, PKC, PLC, PSD-95, syb-2 and c-Fos were detected by western blot. After administration of G15 for 0.5 h, the head withdrawal threshold was determined. Sacrificed the rats, and the RVM tissue was isolated and homogenized. Measuring the protein concentrations. The proteins were added to sodium dodecyl sulfate-polyacrylamide gel electrophoresis (SDS-PAGE) gel electrophoresis for 1.5 h (Beyotime, Shanghai, China), and then transferred to polyvinylidene difluoride (PVDF) membranes (Millipore, USA). The membranes were sealed with 5% skim milk for 1 h at 37°C and incubated with primary antibodies, including anti-GluA1 (1:1,000, Cell Signaling Technology, Danvers, Massachusetts, USA), anti-GPER1 (1:1,000, Thermo Fisher Scientific, 5,823 Newton Drive, Carlsbad, USA), anti-PKC (1:1,000, Abcam, UK), anti-PLC (1:1,000, Abcam, Cambridge, UK), anti-c-Fos (1:1,000, Affinity, Jiangsu, China), anti-PSD-95 (1:1,000, Cell Signaling Technology, Danvers, Massachusetts, USA), anti-syb-2 (1:1,000, Abcam, Cambridge, UK) and anti-GAPDH (1:10,000, ZEN-Bioscience, Chengdu, China), overnight at 4°C. Membranes were washed with Tris-buffered saline containing Tween 20 (TBST) and incubated with the corresponding secondary antibodies (1,10,000, Mengbio, Chongqing, China) for 2 h at 37°C. The blots were detected using a detection kit (Beyotime, Shanghai, China) and visualized using an imaging system (Bio-Rad, Hercules, California, USA). GAPDH was used as a loading control to normalize protein levels. We used stripping buffer (Solarbio, Beijing, China) to remove the membrane-bound antibody. The next round of western blot experiments was then performed using a different antibody, and using this strategy enabled us to detect proteins on the same membrane multiple times.

### 2.8. Immunofluorescence staining

Rats were perfused with 0.9% saline and 4% paraformaldehyde *via* the heart after injecting inhibitor or activator. The brain stem area was immediately separated, fixed in 4% paraformaldehyde for 24 h, and then soaked in sucrose solution with increased concentration (20–30%) in turn until the tissue sank to the bottom. RVM sections were cut into 10 μm-thick sections using a frozen sectioning machine (Leica CM1950, Wetzlar, Germany) and stored at −80°C for subsequent use. Sections were removed from the −80°C refrigerator, brought to room temperature, and then repaired with the EDTA antigen retrieval solution (PH 8.0, Solarbio, Beijing, China) in a microwave oven at a low temperature (approximately 40°C) for 10 min. Next, the slices were cooled to room temperature and washed thrice with phosphate-buffered saline (PBS; Biosharp, Anhui, China), and 0.3% TritonX-100 (Mengbio, Chongqing, China) was added. After incubation at 37°C for 10 min, the slices were washed again with PBS thrice; then, 5% goat serum (Solarbio, Beijing, China) was added. The slices were then incubated at 37°C for a further 30 min. The slices were then incubated at 4°C overnight using anti-neuronal nuclei (NeuN; 1:200, host mouse, Cell Signaling Technology), anti-GFAP (1:200, host mouse, Cell Signaling Technology), anti-Iba1 (1:200, host mouse, Abacam), anti-GPER1 (1:200, host rabbit, Thermo Fisher) and anti-c-Fos (1:200, host mouse, Abcam) antibodies. After washing thrice with PBS, the sections were incubated with fluorescently coupled secondary antibodies (Alexa Fluor 488-labeled goat anti-rabbit IgG and Alexa Fluor 596-labeled goat anti-mouse IgG, ZSGB-Bio, Beijing, China) at 37°C for 90 min. Micrographs were analyzed using a fluorescence confocal microscope (EU 5888; Leica, Wetzlar, Germany). Three sections were randomly selected per rat. Images of cells were captured using a 20× objective. The immunoreactivity of c-Fos and GPER1 in the RVM was captured using a 10× objective. The number of positive cells was calculated as the average number of positive cells obtained from three images.

### 2.9. Transmission electron microscopy

Following anesthesia, the rats were perfused with 2.5% glutaraldehyde and 2% paraformaldehyde mixed liquid, and their brains were taken out. The RVM was separated and fixed in 4% glutaraldehyde at 4°C for 24 h. The RVM was cut into 1 × 1 × 1 mm tissue blocks, fixed, embedded, sectioned, and stained. Refer to this document for specific methods (Wang et al., 2021). Images were captured using a JEM-1400Plus transmission electron microscope (TEM; JEOL Ltd., Tokyo, Japan) and analyzed using Image Pro Plus 6.2. Synaptic morphological parameters were measured at 50,000× magnification. The width of the synaptic space and the thickness of the postsynaptic density (PSD) were measured using the multipoint average method and Güldner and Ingham’s method (Güldner and Ingham,1980).

### 2.10. Data analysis

The data are presented as the mean ± standard deviation (SD) and were statistically analyzed using GraphPad Prism 8.2.1 (San Diego, CA, USA). The Photoshop software (Adobe Photoshop 2021) was used to generate the illustrations. The results of behavioral tests were analyzed with two-way repeated measure analysis of variance (ANOVA) followed by the Bonferroni *post hoc* test. One-way ANOVA analysis was followed by the Tukey *post hoc* test for multiple group comparisons. Differences between two groups were examined using independent-sample t-tests. *p*-value <0.05 are considered significant differences.

## 3. Results

### 3.1. CFA rats show a significant decrease in the mechanical pain threshold and an upregulation in the expression of c-Fos and GPER1

**Figure 1:**
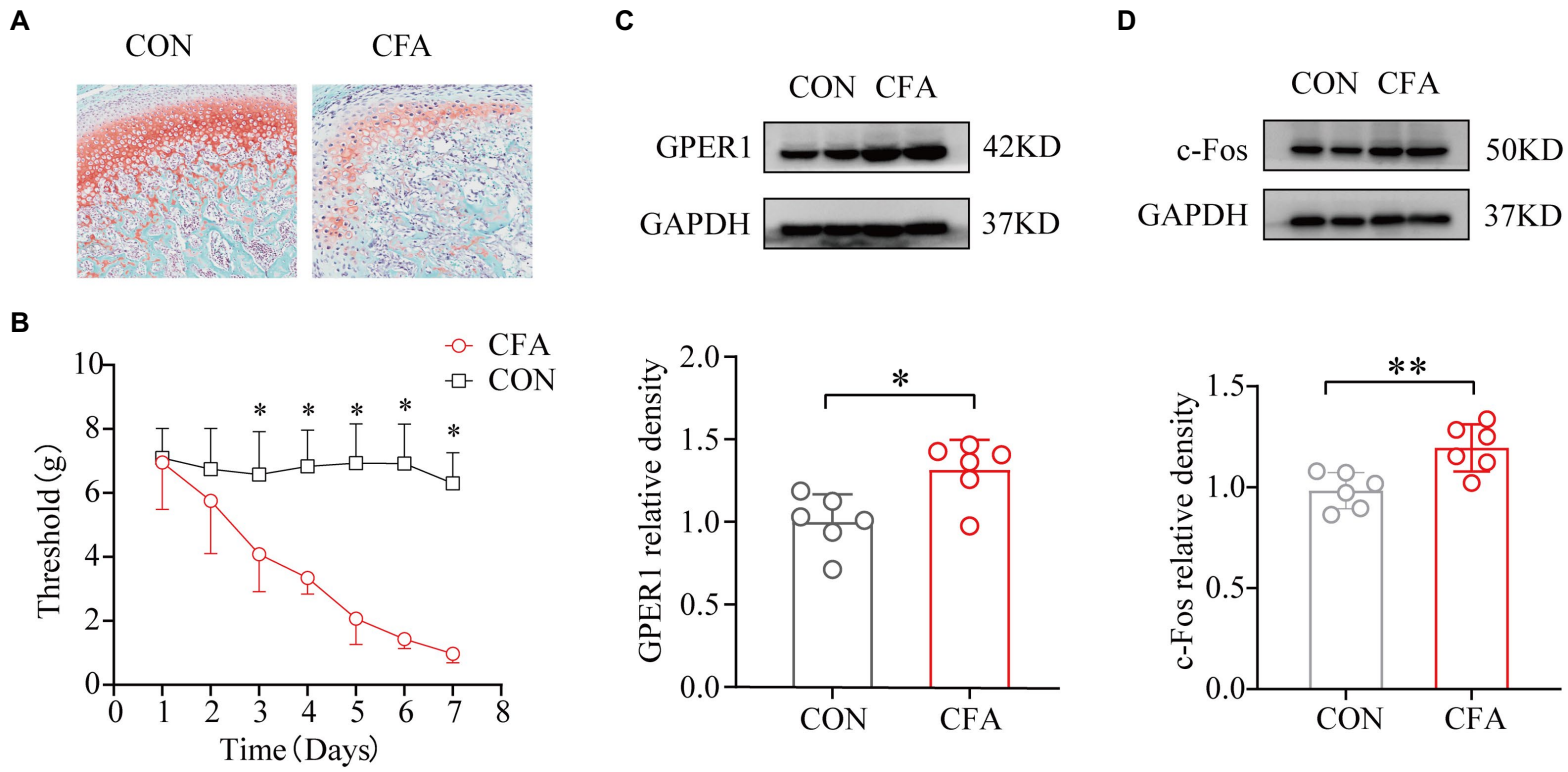
Development of mechanical heterotopic pain, GPER1, and c-Fos expression increased in RVM of CFA-induced chronic pain model. Morphological characteristics of the temporomandibular joint cartilage in rats. **(A)** Articular cartilage staining of cartilage tissues with red solid green showed a significant loss of proteoglycan in the CFA group compared with the CON group, indicating significant bone destruction of the tissue. **(B)** Determination of orofacial pain threshold in rats. Development of mechanical ectopic pain in rats. a Significantly lower orofacial pain thresholds were significantly lower 3 days after CFA injection compared with 3 days after NS injection. **(C, D)** WB assay of GPER1 and c-Fos expression in RVM showed that GPER1 and c-Fos protein levels were upregulated in the CFA group compared with the CON group. Quantification of normalized WB experiments showed a significant increase in the CFA group. All data were normalized to the GAPDH control group. Data represent the mean ± SD. **(B)** was measured with two-way repeated ANOVA followed by the Bonferroni *post hoc* test. A significant decrease in the thresholds was observed after 3 days of NS infusion compared with those detected after 3 days of CFA infusion. **(C, D)** were measured with statistical analysis was performed using independent samples t-test. *p < 0.05, **p < 0.01. *n* = 6 per group. Compared with the CON group.

Intense erythema and damage to the articular cartilage in the TMJ region were observed in the CFA-injected rats but not in the CON rats (Figures 1A,B). We used the von Frey hair to determine the head withdrawal threshold. The rats injected with 0.9% normal saline served as the CON group. Before injection, no difference was observed in the head withdrawal threshold between the two groups. The head withdrawal thresholds in the CON group did not change significantly with time, while the thresholds in the orofacial region showed a significant decrease in the CFA group from day 3 onwards. Additionally, the head withdrawal threshold of rats in the CFA group decreased significantly compared with those of rats in the CON group on day 7 (Figure 1C). The expression of c-Fos and GPER1 in the RVM increased significantly after CFA injection (Figures 1D,E). These results showed that GPER1 may be involved in the pathophysiological mechanism of chronic orofacial pain, while the upregulation of c-Fos suggests the activation of neurons in RVM region.

### 3.2. G15 injection causes a downregulation in the expression of GPER1 and c-Fos in the RVM

**Figure 2:**
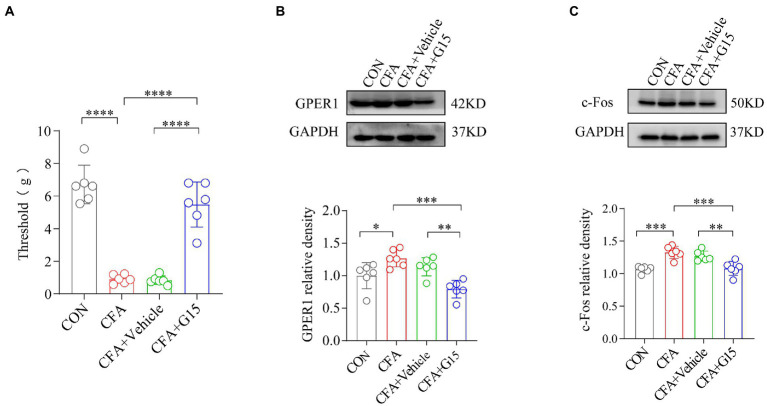
G15 attenuated CFA induced pain by reducing c-Fos and GPER1 expression. **(A)** Orofacial pain thresholds in rats. Compared with the CFA group, the orofacial pain thresholds of rats in the CFA + G15 group were significantly upregulated, and there was no significant difference between the CFA group and CFA + Vehicle group. WB detection of GPER1 and c-Fos expression in RVM. GPER1 **(B)** and c-Fos **(C)** protein levels were down-regulated in the CFA + G15 group compared with the CFA group, and there was no significant difference between the CFA group and CFA + Vehicle group. All data were normalized to the GAPDH control group. All statistical analysis was performed using one-way ANOVA followed by the Tukey *post hoc* test for multiple group comparisons. *p < 0.05, **p < 0.01, ***p < 0.001, ****p < 0.0001. *n* = 6 per group.

The head withdrawal threshold of rats in the CFA group was reduced 1 week after the bilateral TMJ cavity injection of CFA. To investigate the role of GPER1 in the chronic pain of the orofacial region, we injected G15 intracerebrally in CFA rats. In accordance with previous studies, we found that G15 produced a significant inhibitory effect 30 min after injection (An et al., 2014). Therefore, the RVM of the rats was taken immediately after measuring the orofacial pain threshold 30 min after G15 injection, and the expression of GPER1 and c-Fos was analyzed by protein blotting. The results show that, compared with CFA rats, rats which underwent G15 injection had significantly increased pain thresholds (Figure 2A). Moreover, the protein blotting results suggest that the expression levels of GPER1 and c-Fos in the body were significantly higher in the CFA group than in the CON and CFA + G15 groups (Figures 2B,C). We did not observe significant difference between the CFA and CFA + Vehicle groups, which suggests that the 0.1% DMSO did not change the levels of GPER1 and c-Fos.

### 3.3. G15 injection alleviates central sensitization induced by CFA

**Figure 3:**
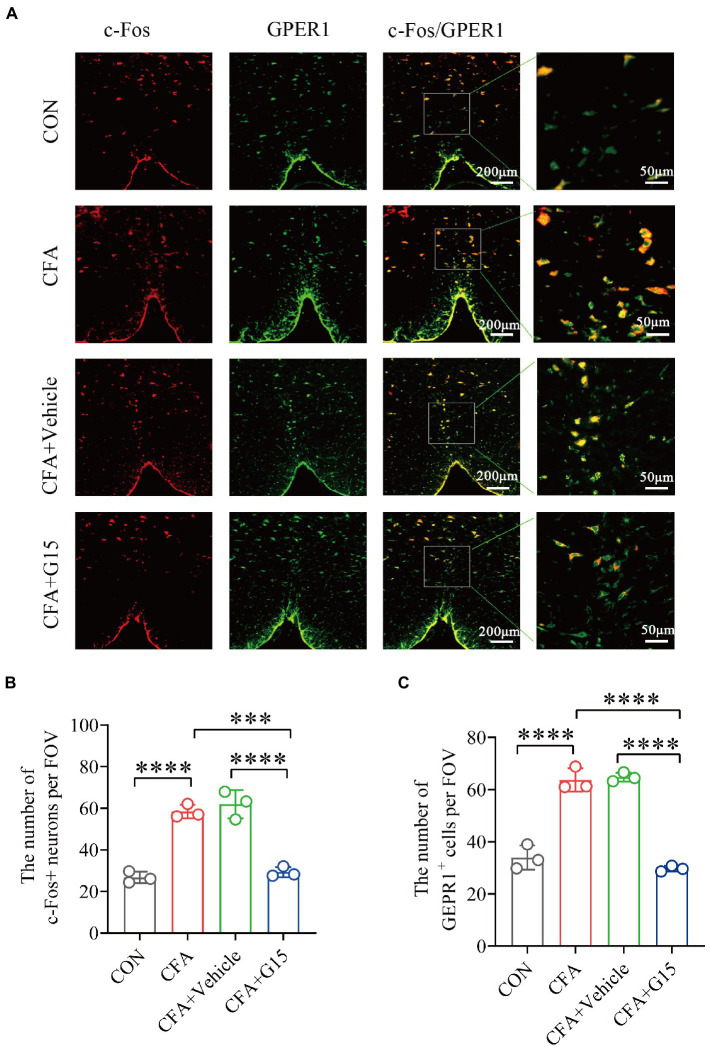
Number of activated GPER1-positive and c-Fos-positive cells in the RVM under different pain states. **(A)** Representative immunofluorescence images showing the distribution of c-Fos^+^ and GPER1^+^ cells in the RVM of different pain states. White arrows show the c-Fos/GPER double positive (c-Fos^+^/GPER1^+^) cells. Red: c-Fos^+^ neurons. Green: GPER^+^ cells. Yellow: Indicates integrated signal. **(B)** Number of c-Fos neurons in the RVM of rats in different states. The number of c-Fos positive neurons was significantly reduced in the CFA + G15 group compared with the CFA group, while there was no significant difference between the CFA group and CFA + Vehicle group. **(C)** Statistical analysis of GPER1^+^ cells in the RVM of rats in different states. The number of GPER1 positive cells was significantly increased in the CFA group compared with the CON group. It was no significantly difference between the CFA group and CFA+ Vehicle group. Data represent the mean ± SD. All statistical analysis was performed using one-way ANOVA followed by the Tukey *post hoc* test for multiple group comparisons. ***p < 0.001, ****p < 0.0001. *n* = 3 per group.

c-Fos, a marker of neuronal activation, has been recognized as a reliable marker for central sensitization. The gene c-Fos and its protein product were reported by Hunt et al., and used to study nociception (pain; Harris, 1998; Marvaldi et al., 2020). c-Fos can be used as a cross synaptic marker of neuronal activity after harmful stimulation (Bullitt, 1990). Immunofluorescence results showed that the number of c-Fos-positive (c-Fos^+^) neurons in the RVM of rats in the CFA group was significantly higher than that of rats in the CON and CFA + G15 groups (Figure 3B). The number of GPER1-positive cells was significantly higher in the CFA group than in the CON and CFA + G15 groups (Figure 3C). These results indicate that G15 alleviated the central sensitization of CFA rats. We did not observe significant difference between the CFA and CFA + Vehicle groups.

### 3.4. The changes of Iba1 positive cells in RVM

**Figure 4:**
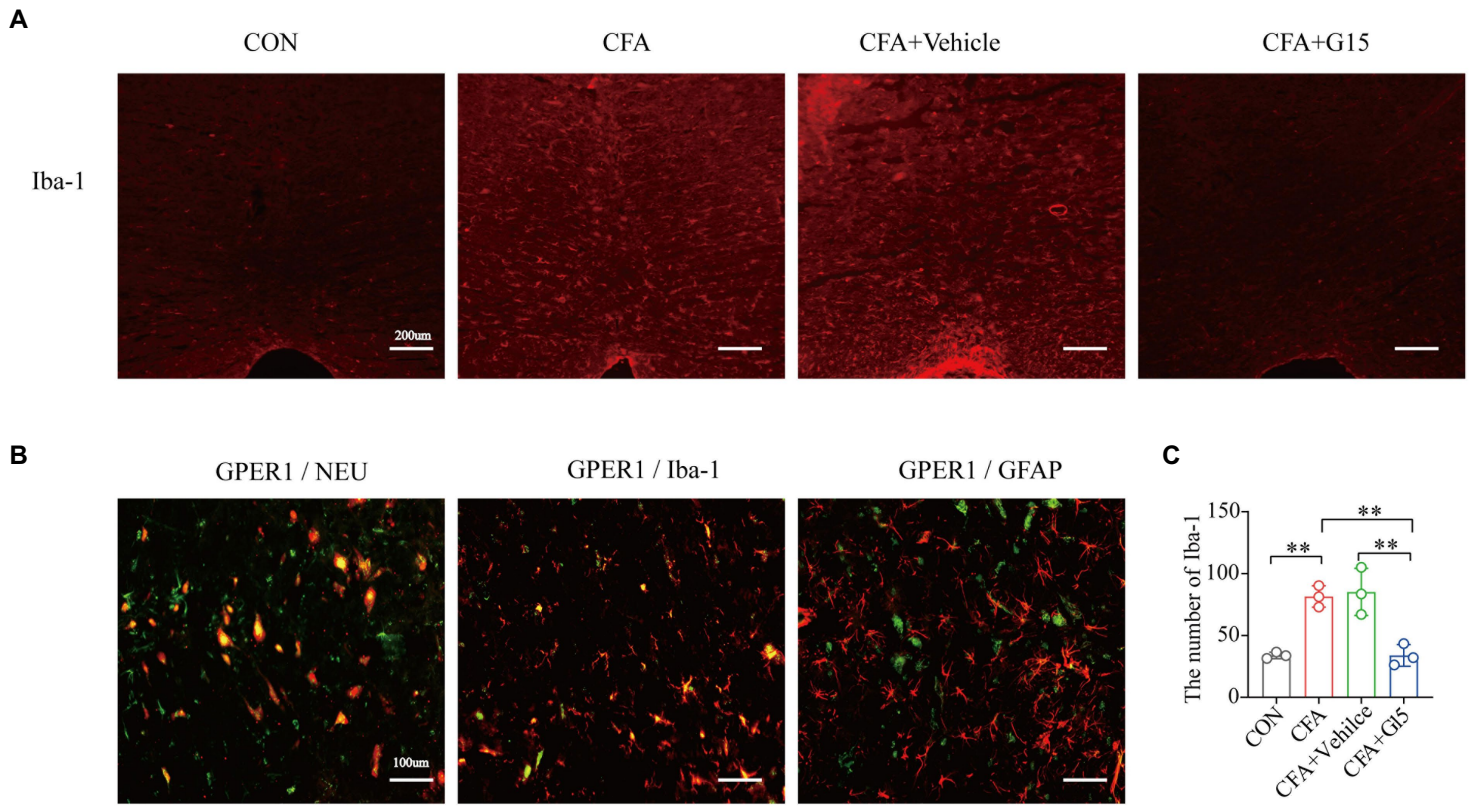
Changes of Iba-1 positive cells in RVM. Number of activated Iba-1-positive cells in the RVM under different pain states. **(A)** Representative immunofluorescence images showing the distribution of Iba1 labeled microglia in the RVM of different pain states. Red: microglia. **(B)**. GPER1 was mainly expressed in NeuN-labeled neurons in Iba 1 labeled microglia, but not in glial fibrillary acidic protein (GFAP)-labeled astrocytes at CON group. Red: Iba1 labeled microglia, Neu labeled neurons, glial fibrillary acidic protein (GFAP) labeled astrocytes. Green: GPER1^+^ cells. Yellow: Indicates integrated signal. **(C)** Number of Iba1 labeled microglia in the RVM of rats in different states. The number of Iba1 labeled microglia was significantly reduced in the CFA + G15 group compared with the CFA group, while there was no significant difference between the CFA group and CFA + Vehicle group. Data represent the mean ± SD. All statistical analysis was performed using one-way ANOVA followed by the Tukey *post hoc* test for multiple group comparisons. **p < 0.01. *n* = 3 per group.

Immunofluorescence results show that the number of Iba1-positive cells in the RVM of rats in the CFA group was significantly higher than that of rats in the CON and CFA + G15 groups (Figures 4A,C). We did not observe significant difference between the CFA and CFA + Vehicle groups. Additionally, it was observed that GPER1 is expressed in Iba1 labeled microglia and Neu labeled neurons, but not in glial fibrillary acidic protein (GFAP) labeled astrocytes (Figure 4B).

### 3.5. G15 injection decreases the expression of GluA1 in the RVM of CFA rats, and GluA1 co-localization in microglia

**Figure 5:**
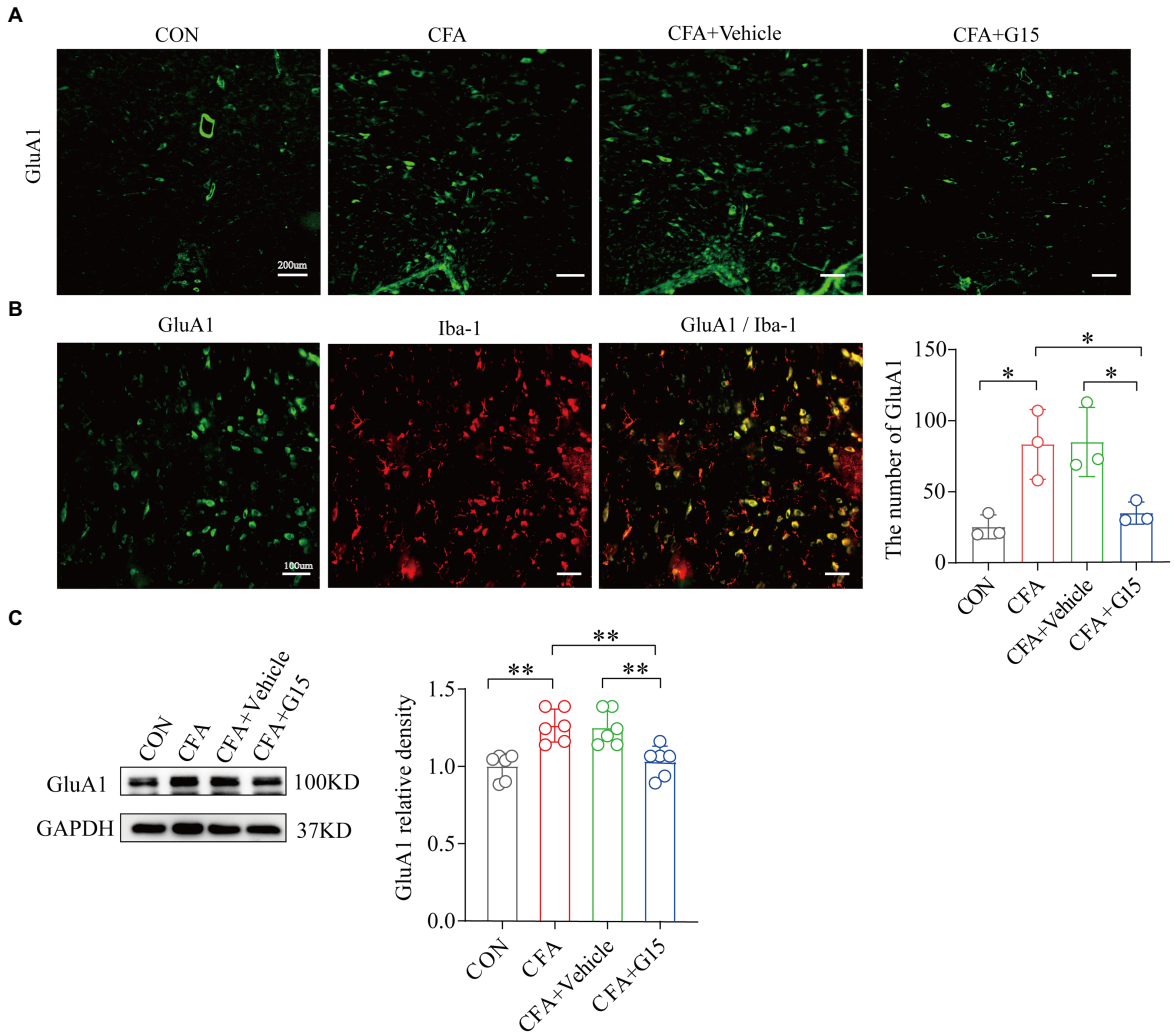
Expression of GluA1 and GluA1 co-localization in microglia. **(A)** Representative immunofluorescence images showing the distribution of Iba1 labeled microglia in the RVM of different pain states. **(B)** In CFA group, GluA1 was expressed in iba 1 labeled microglia. **(C)** Number of GluA1 in the RVM of rats in different states. The number of GluA1 was significantly reduced in the CFA + G15 group compared with the CFA group, while there was no significant difference between the CFA group and CFA + Vehicle group (*n* = 3 per group). WB detected the expression of GluA1**(D)** in RVM after G15 injection, and the expression of GluA1 protein was decreased in the CFA + G15 group compared with the CFA group (*n* = 6 per group). There was no significant difference between the CFA group and the CFA + Vehicle group. Data represent the mean ± SD. All data of WB were normalized to the GAPDH control group. Statistical analysis was performed using one-way ANOVA followed by the Tukey *post hoc* test for multiple group comparisons. *p < 0.05, **p < 0.01.

To determine whether G15 influences α-amino-3-hydroxy-5-methyl-4-isoxazole propionic acid (AMPA) receptor expression (Figure 5A), we measured the protein expression of total GluA1 by western blotting (Figure 5C). Our results show that the expression of GluA1 in the CFA group was significantly increased compared to the CON group. G15 administration significantly downregulated total GluA1 levels and the phosphorylation of GluA1. We did not observe significant difference between the CFA and CFA + Vehicle groups (Figure 5B).

### 3.6. G15 injection reduces the overexpression of the synapse-associated proteins PSD-95 and syb-2 in CFA rats

**Figure 6:**
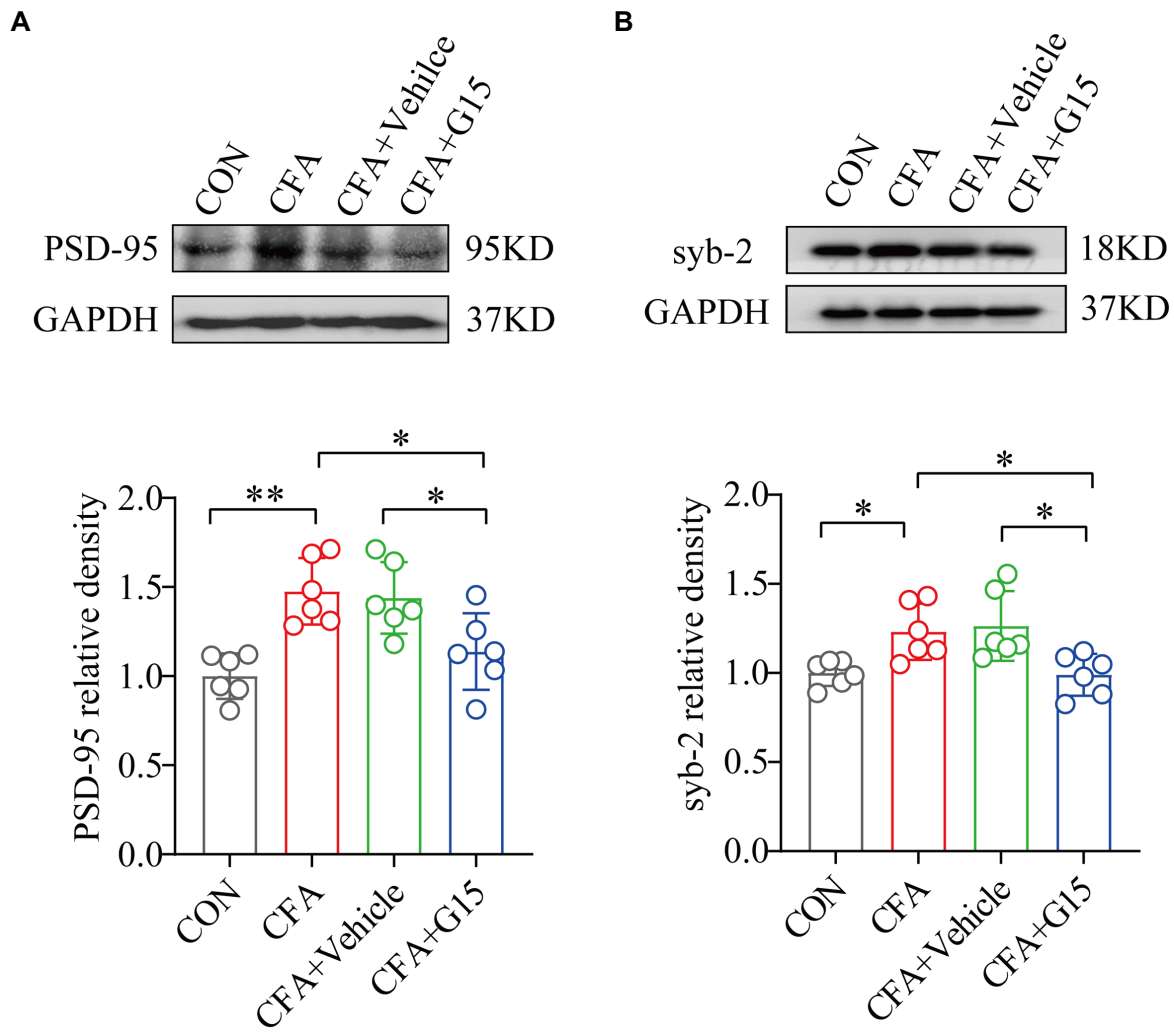
Effect of G15 on synapse-associated protein expression levels. Representative WB bands showing the expression of PSD-95 **(A)** and syb-2 **(B)**. WB detection of PSD-95 and syb-2 expression in RVM. PSD-95 **(A)** and syb-2 **(B)** protein levels were down-regulated in the CFA + G15 group compared with the CFA group, and there was no significant difference between the CFA group and CFA + Vehicle group. All data were normalized to the GAPDH control group. All statistical analysis was performed using one-way ANOVA followed by the Tukey *post hoc* test for multiple group comparisons. *p < 0.05, **p < 0.01. *n* = 6 per group.

Since GluA1 regulates synaptic plasticity, which is crucial in the development of chronic orofacial pain, we explored whether G15 regulates the expression of two synaptic-associated proteins; PSD-95 and syb-2 (Figures 6A,B). Western blotting showed that the expressions of PSD-95 and syb-2 in the RVM region of CFA group rats were significantly increased. Injection of G15 reduced the expression of these proteins. We did not observe significant difference between the CFA and CFA + Vehicle groups.

### 3.7. G15 injection restores aberrant synaptic plasticity in CFA rats

**Figure 7:**
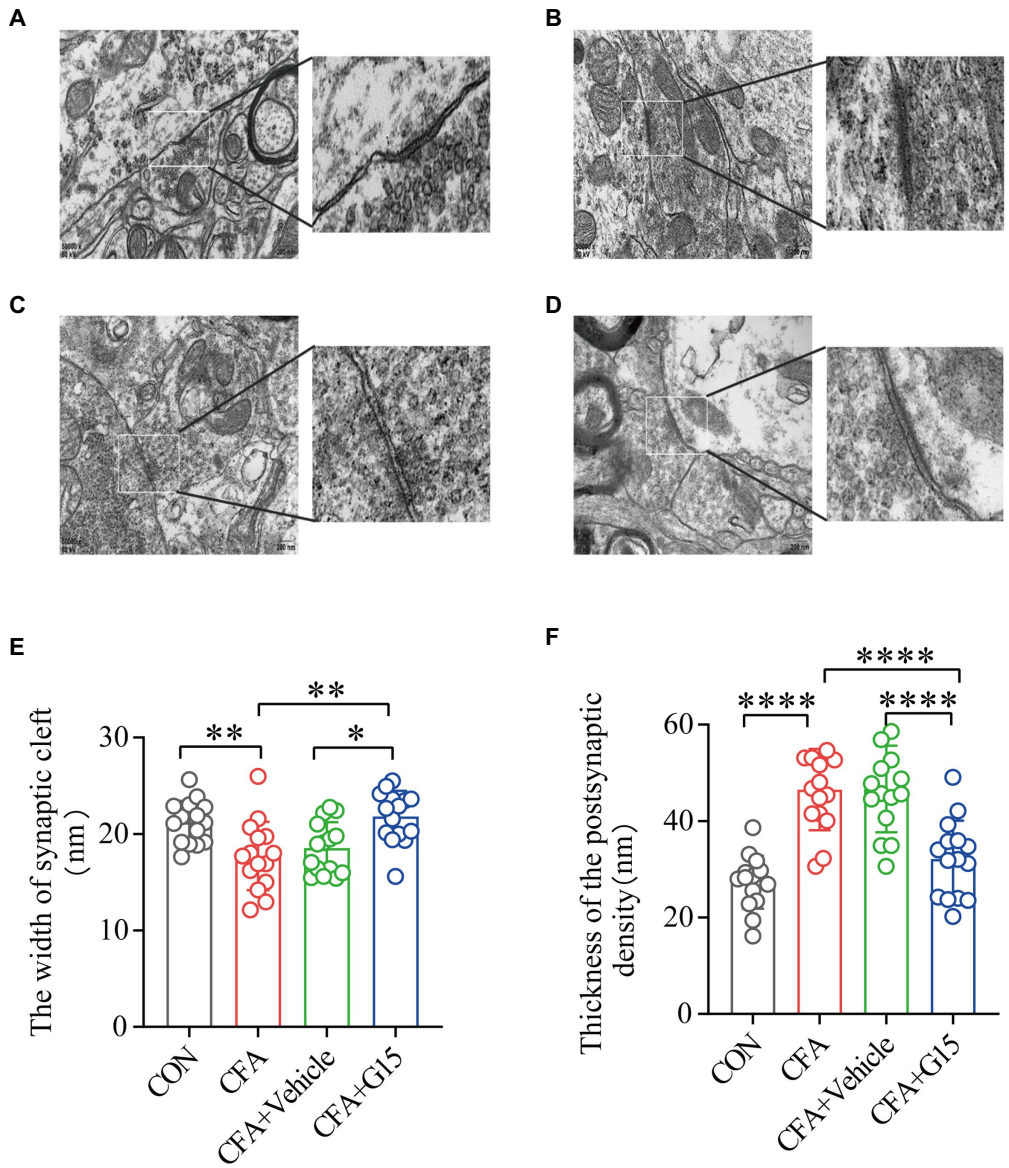
Synaptic structure of the RVM neurons in each group. **(A)** CON group; **(B)** CFA group; **(C)** CFA + Vehicle group; **(D)** CFA + G15 group. (**E**) The width of the synaptic cleft was decreased in the CFA group and CFA + Vehicle group compared with the CON group. There was no significant difference between the CFA group and the CFA + Vehicle group. (**F**) The thickness of PSD was significantly increased in the CFA group and CFA + Vehicle group compared with the CON group. There was no significant difference between the CFA group and the CFA + Vehicle group. Data represent the mean ± SD. Statistical analysis was performed using one-way ANOVA followed by the Tukey *post hoc* test for multiple group comparisons. *p < 0.05, **p < 0.01, ****p < 0.0001. *n* = 3 per group.

Changes in synaptic structure and function are closely related to synaptic plasticity (10.6084/m9.figshare.20445930). Representative images of synaptic structures in the RVM were observed using TEM (Figure 7). Synaptic spaces were visible in the CON and CFA + G15 groups, and the PSD was appropriate (Figures 7A,D). Conversely, the synaptic space and PSD of the CFA + Vehicle and CFA groups were contracted and blurred (Figures 7B,C). The results displayed that, compared with that in the CON group, the width of the synaptic clefts in the CFA and CFA + Vehicle groups was decreased (Figure 7E). The PSD thickness of the CFA group was significantly greater than that of the CON group. After G15 treatment, these abnormal changes were alleviated (Figure 7F). These results also provide intriguing, albeit indirect, evidence for the mechanism of GPER1 involvement in chronic orofacial pain, which may be related to local sensitization in the RVM region.

### 3.8. GPER1 induces nociceptive hyperalgesia *via* the PLC-PKC signaling pathway

**Figure 8:**
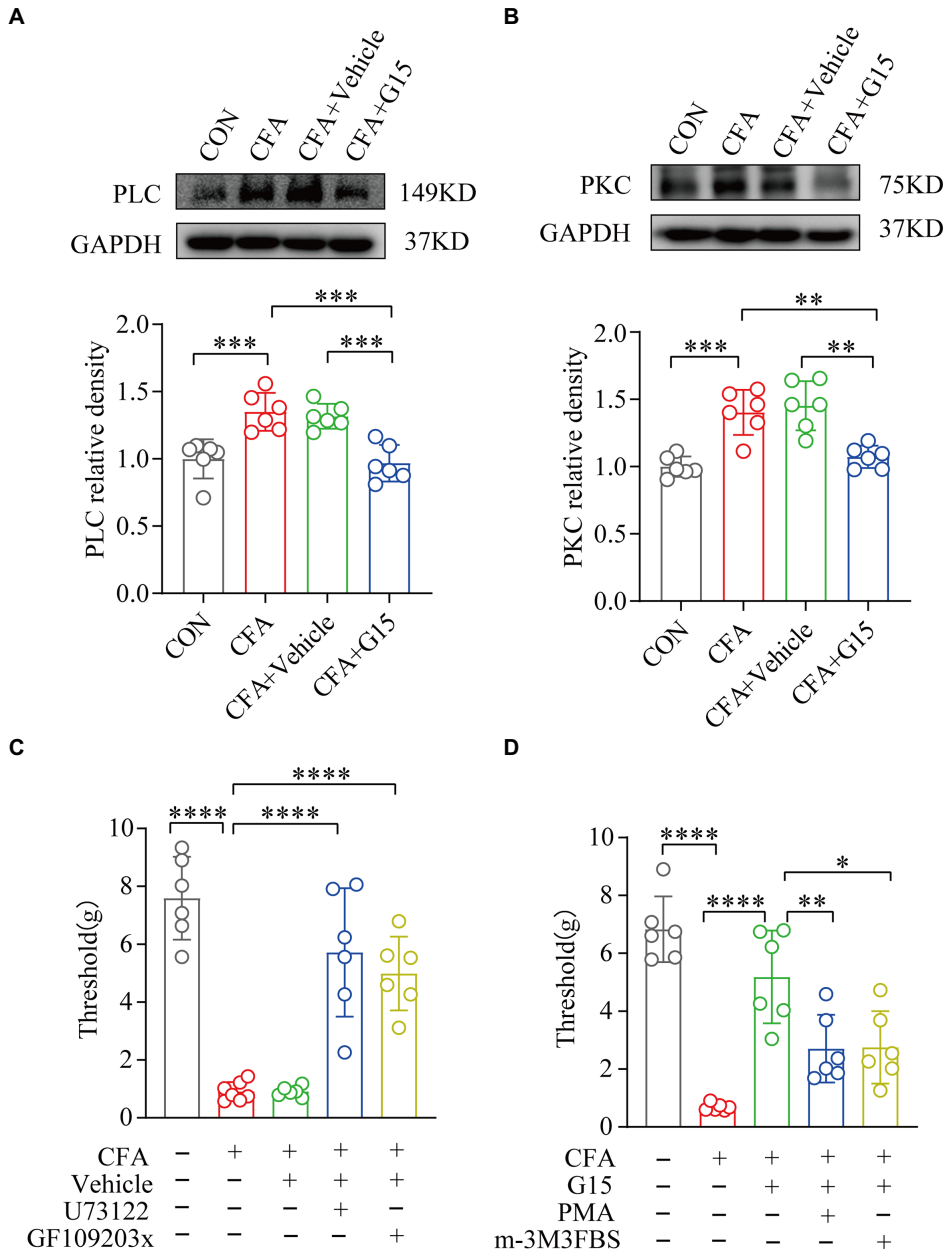
G15 alleviated CFA-induced pain through the PLC-PKC signaling pathway. **(A, B)** WB detection of PLC and PKC expression in RVM. The levels of these proteins were significantly different in both the CON and CFA + G15 groups compared with the CFA group. There was no significant difference between the CFA group and CFA + Vehicle group. **(C)** Treatment with GF109203x and U73122 significantly reversed the effect of G15 compared with the CFA + G15 group. There was no significant difference compared with the G15 + Vehilce group. **(D)** Treatment with m-3M3FBS and PMA significantly increased orofacial pain thresholds compared with the CFA group, with no significant difference compared with the CFA + Vehicle group. All statistical analysis were performed using one-way ANOVA followed by the Tukey *post hoc* test for multiple group comparisons. *p < 0.05, **p < 0.01, ***p < 0.001, ****p < 0.0001. *n* = 6 per group.

In this study, we found that PLC and PKC protein expression was significantly higher in the RVM of rats in the CFA group compared with that of rats in the CON group, while G15 injection significantly downregulated their expression in the RVM of CFA rats (Figures 8A,B). There was no significant difference between the CFA and CFA + Vehicle groups. Rats in the CFA group were randomly selected and divided into a CFA + PLC inhibitor (U73122), CFA + PKC inhibitor (GF109203x), and a CFA + vehicle group, and the pain thresholds of the rats were measured 1 day after drug injection. Compared with the CFA group rats, the CFA + PLC and CFA + PKC inhibitor group rats showed a significant increase in pain thresholds (Figure 8C). Similarly, rats in the G15 group were randomly selected and divided into a G15 + PLC agonist group (m-3M3FBS) and a G15 + PKC agonist group (PMA), and the withdrawal threshold level of the rat heads was measured 1 day after drug injection. Compared with the G15 group rats, G15 + PLC and G15 + PKC agonist group rats showed significantly increased pain thresholds as well (Figure 8D). Therefore, we speculate that GPER1 may cause a decrease in the orofacial mechanical pain threshold in CFA rats through the PLC-PKC signaling pathway.

## 4. Discussion

The chronification of acute pain is receiving increasing attention (Jackson et al., 2015). Many studies have proved that the RVM region plays a key role in the transition from acute postoperative pain to chronic pain, and in the downstream pain regulation system (Xu et al., 2021). However, orofacial pain is different from somatic pain because there are many important adjacent structures in the oral and facial region, which makes it easier for patients with chronic oral and facial pain to experience negative exacerbations (such as affected sleep, cognition, and memory which further complicate the pain. Our experiments explored the mechanism of the role of the RVM in the chronification of acute pain in the orofacial region. In this study, we used bilateral CFA temporomandibular joint cavity injections to simulate the transition from acute inflammatory pain to chronic pain in the orofacial region (Araújo-Filho et al., 2018). The current study provides convincing evidence that RVM cells are significantly activated in persistent orofacial pain, consistent with the onset of chronic pain. Previous experiments have shown that GPER1^+^ cells in the RVM can modulate the chronicity of postoperative somatic pain (Xu et al., 2021). The same results were obtained in our experiment and the pain was relieved after GPER1 inhibitor injection, suggesting that GPER1 is also involved in the chronicity of orofacial pain.

Persistent peripheral injurious stimuli may cause central sensitization, which may be one of the mechanisms of hyperalgesia. Several important brain nuclei have been shown to be involved in central sensitization during the onset of chronic migraine and somatic neuropathic pain. The RVM consists of the adjacent reticular formation and the large nucleus of the middle suture and is considered a key player in the downstream pain modulation system. Many different nuclei transmit information to RVM, which is considered as the key role of downstream pain regulation system (Chen and Heinricher, 2019). The downstream PAG-RVM pathway regulates the upstream injurious transmission of spinal dorsal cord and participates in the development of chronic pain (Lau and Vaughan, 2014). Previous studies have shown that synaptic gaps and changes in synaptic plasticity-related proteins are necessary for central sensitization (Wang et al., 2021). Our results found that during chronic orofacial pain a narrowing of the gap between synapses in the RVM region occurs, and this along with changes affecting synapse-associated plasticity proteins (PSD-95 and syb-2) suggest that central sensitization of the RVM region may be involved in the chronicity of orofacial pain. This is consistent with the study conducted by Mo et al. (2022).

It was shown that there are two main types of cells expressing GPER1 in the RVM region: 1. Non-5-hydroxytryptaminergic, mainly GABAergic (Winkler et al., 2006; Pedersen et al., 2011; Gao et al., 2021b). Injurious stimulation activates GABAergic neurons in the RVM, inhibiting dorsal horn enkephalins and GABAergic interneurons from promoting mechanical pain (François et al., 2017). In addition, RVM can also project to the medullary dorsal horn (MDH) to regulate the hyperalgesia in the trigeminal innervation area, which may be related to the enhanced downward facilitation of RVM (Chai et al., 2012; Mo et al., 2022). 2. Neuroglia (Kuo et al., 2010; Lee et al., 2012; Fuente-Martin et al., 2013), which can induce the formation of dendritic synapses through local release of glutamate (McCarthy et al., 2002; Brailoiu et al., 2007). Interestingly changes in GluA1 in the RVM region were found in this study as well as other previous studies on chronic pain (Guan et al., 2003, 2004; Radhakrishnan and Sluka, 2009). Meanwhile, our results showed that RVM microglia was activated simultaneously in chronic orofacial pain induced by CFA. However, double immunofluorescence staining showed that GPER1 co-located with microglia and neurons rather than astrocytes. This is consistent with previous findings (Correa et al., 2020; Xu et al., 2022). Among them, the activation of microglia GPER1 induces the release of glutamate to facilitate chronic pain. This mechanism may be related to the involvement of microglia in synaptic plasticity (Hagino et al., 2004; Matute et al., 2006). In this study, we found that microglia co expressed with GluA1 in RVM, which further explained the effect of microglia on synapses. The expression of functional glutamate receptor(GluR)on microglia in the developing and mature brain has been confirmed (Murugan et al., 2013). Based on these results, we believe that GPER and microglia in RVM actively participate in chronic orofacial pain. This process is related to the synaptic plasticity of RVM neurons. An electrophysiological study showed that CFA induced persistent inflammatory pain could enhance AMPA receptor mediated excitatory postsynaptic current in nucleus raphe magnus (an important component of RVM; Tao et al., 2014). In addition, our study found that GPER1 activated by chronic orofacial pain co-located with RVM neurons. GPER1 in RVM contributes to postoperative pain chronicity and GPER1 localization in RVM is associated with GABAergic neurons (Gao et al., 2021a). In this study, we confirmed the positive synergistic effect of GPER1 and microglia in RVM on chronic orofacial pain. Here, we noticed that the RVM region expressed GABAergic neurons, but in our study, both western blot and immunofluorescence showed that there was glutamate receptor in RVM. This conclusion suggests that glutamate receptor probably originates from microglia, and the glutamate produced is taken up by nearby astroglia and transformed into glutamine, which is crucial for neurons to synthesize GABA (Héja et al., 2019; Kruyer et al., 2023).

It is reported that GPER1 can bind to the G-coupled protein alpha subunit and activate PLC (Cheng et al., 2016). Activated PLC leads to PKC production and intracellular calcium mobilization, which mediates the phosphorylation of GluA1 (An et al., 2014). Kuhn et al. demonstrated that GPER1 mediates the rapid action of estrogen on injurious neurons, producing mechanical nociceptive hyperalgesia and activating PKCƐ (Kuhn et al., 2008). In our study, PLC-PKC expression was significantly increased at the onset of chronic pain in the orofacial region, and administration of U73122 or GF109203x injections (PLC-PKC inhibitors) relieved chronic pain in the orofacial region. In contrast, rats in the CFA + G15 group receiving m-3M3FBS or PMA (PLC-PKC agonists) could significantly reverse the effect of G15, causing the pain threshold to decrease again. Thus, the involvement of PLC-PKC in the development of chronic pain throughout the orofacial region was confirmed.

Our results provide strong evidence that persistent inflammatory pain in the orofacial area is associated with GPER1 activation of RVM microglia. The underlying mechanism may be central sensitization mediated by microglia activation in the RVM area, where GluA1 and PKC-PLC are involved in this process.

## 5. Conclusion

Our findings provide strong evidence that GPER1 in RVM is associated with chronic pain in the orofacial region. Persistent oral and facial inflammatory pain stimulates GPER1 on microglia in the RVM region, increases synaptic plasticity mediated by PLC-PKC pathway, and further leads to the formation of chronic orofacial pain. These results suggest that GPER1 and microglia is a potential target for the prevention and treatment of chronic orofacial pain.

## Data availability statement

The datasets presented in this study can be found in online repositories. The names of the repository/repositories and accession number(s) can be found in the article/supplementary material.

## Ethics statement

The animal study was reviewed and approved by the Ethics Committee of the Stomatology Hospital affiliated Chongqing Medical University (registration no. CQHS-NT022-2022).

## Author contributions

CY, JZ, and WZ conceived of and designed the study. JW collected the data. SL and XH participated in data analysis and interpretation. WZ drafted the manuscript and FG and ZC critically revised the manuscript. All authors contributed to the article and approved the submitted version.

## Funding

This work was supported by CSA Clinical Research Fund (CSA-A2021-05) and the joint project of Chongqing Health Commission and Science and Technology Bureau Chongqing, China (Grant No. 2021MSXM188).

## Conflict of interest

The authors declare that the research was conducted in the absence of any commercial or financial relationships that could be construed as a potential conflict of interest.

## Publisher’s note

All claims expressed in this article are solely those of the authors and do not necessarily represent those of their affiliated organizations, or those of the publisher, the editors and the reviewers. Any product that may be evaluated in this article, or claim that may be made by its manufacturer, is not guaranteed or endorsed by the publisher.

## References

[ref1] Akama K. T. Thompson L. I. Milner T. A. McEwen B. S. (2013). Post-synaptic density-95 (PSD-95) binding capacity of G-protein-coupled receptor 30 (GPR30), an estrogen receptor that can be identified in hippocampal dendritic spines. J. Biol. Chem. 288, 6438–6450. doi: 10.1074/jbc.M112.412478, PMID: 23300088 PMC3585078

[ref2] An G. Li W. Yan T. Li S. (2014). Estrogen rapidly enhances incisional pain of ovariectomized rats primarily through the G protein-coupled estrogen receptor. Int. J. Mol. Sci. 15, 10479–10491. doi: 10.3390/ijms150610479, PMID: 24921706 PMC4100163

[ref3] Araújo-Filho H. G. Pereira E. W. M. Campos A. R. Quintans-Júnior L. J. Quintans J. S. S. (2018). Chronic orofacial pain animal models - progress and challenges. Expert Opin. Drug Discov. 13, 949–964. doi: 10.1080/17460441.2018.1524458, PMID: 30220225

[ref4] Avivi-Arber L. Sessle B. J. (2018). Jaw sensorimotor control in healthy adults and effects of ageing. J. Oral Rehabil. 45, 50–80. doi: 10.1111/joor.12554, PMID: 28853161

[ref5] Bagley E. E. Ingram S. L. (2020). Endogenous opioid peptides in the descending pain modulatory circuit. Neuropharmacology 173:108131. doi: 10.1016/j.neuropharm.2020.108131, PMID: 32422213 PMC7313723

[ref6] Bai Q. Liu S. Shu H. Tang Y. George S. Dong T.. (2019). TNFalpha in the trigeminal nociceptive system is critical for Temporomandibular joint pain. Mol. Neurobiol. 56, 278–291. doi: 10.1007/s12035-018-1076-y, PMID: 29696511 PMC6698364

[ref7] Brailoiu E. Dun S. L. Brailoiu G. C. Mizuo K. Sklar L. A. Oprea T. I.. (2007). Distribution and characterization of estrogen receptor G protein-coupled receptor 30 in the rat central nervous system. J. Endocrinol. 193, 311–321. doi: 10.1677/JOE-07-0017, PMID: 17470522

[ref8] Bullitt E. (1990). Expression of c-fos-like protein as a marker for neuronal activity following noxious stimulation in the rat. J. Comp. Neurol. 296, 517–530. doi: 10.1002/cne.902960402 2113539

[ref9] Burgess S. E. Gardell L. R. Ossipov M. H. Malan T. P. Jr. Vanderah T. W. Lai J.. (2002). Time-dependent descending facilitation from the rostral ventromedial medulla maintains, but does not initiate, neuropathic pain. J. Neurosci. 22, 5129–5136. doi: 10.1523/jneurosci.22-12-05129.2002, PMID: 12077208 PMC6757729

[ref10] Cao Y. Xie Q.-F. Li K. Light A. R. Fu K.-Y. (2009). Experimental occlusal interference induces long-term masticatory muscle hyperalgesia in rats. Pain 144, 287–293. doi: 10.1016/j.pain.2009.04.029, PMID: 19473767

[ref11] Chai B. Guo W. Wei F. Dubner R. Ren K. (2012). Trigeminal-rostral ventromedial medulla circuitry is involved in Orofacial Hyperalgesia contralateral to tissue injury. Mol. Pain 8:1744-8069-8-78. doi: 10.1186/1744-8069-8-78, PMID: 23092240 PMC3484042

[ref12] Chaplan S. R. Bach F. W. Pogrel J. W. Chung J. M. Yaksh T. L. (1994). Quantitative assessment of tactile allodynia in the rat paw. J. Neurosci. Methods 53, 55–63. doi: 10.1016/0165-0270(94)90144-9, PMID: 7990513

[ref13] Chen Q. Heinricher M. M. (2019). Descending control mechanisms and chronic pain. Curr. Rheumatol. Rep. 21:13. doi: 10.1007/s11926-019-0813-1 30830471

[ref14] Cheng Q. Meng J. Wang X. S. Kang W. B. Tian Z. Zhang K.. (2016). G-1 exerts neuroprotective effects through G protein-coupled estrogen receptor 1 following spinal cord injury in mice. Biosci. Rep. 36:e00373. doi: 10.1042/BSR20160134, PMID: 27407175 PMC5006313

[ref15] Correa J. Ronchetti S. Labombarda F. De Nicola A. F. Pietranera L. (2020). Activation of the G protein-coupled estrogen receptor (GPER) increases neurogenesis and ameliorates Neuroinflammation in the hippocampus of male spontaneously hypertensive rats. Cell. Mol. Neurobiol. 40, 711–723. doi: 10.1007/s10571-019-00766-5, PMID: 31784921 PMC11448800

[ref16] Dubovy P. Klusakova I. Hradilova-Svizenska I. Joukal M. Boadas-Vaello P. (2018). Activation of astrocytes and microglial cells and CCL2/CCR2 Upregulation in the dorsolateral and Ventrolateral nuclei of periaqueductal gray and rostral ventromedial medulla following different types of sciatic nerve injury. Front. Cell. Neurosci. 12:40. doi: 10.3389/fncel.2018.00040, PMID: 29515373 PMC5825898

[ref17] François A. Low S. A. Sypek E. I. Christensen A. J. Sotoudeh C. Beier K. T.. (2017). A brainstem-spinal cord inhibitory circuit for mechanical pain modulation by GABA and Enkephalins. Neuron 93, 822–839.e6. doi: 10.1016/j.neuron.2017.01.008, PMID: 28162807 PMC7354674

[ref18] Fruhstorfer H. Gross W. Selbmann O. (2001). von Frey hairs: new materials for a new design. Eur. J. Pain 5, 341–342. doi: 10.1053/eujp.2001.0250, PMID: 11558991

[ref19] Fuente-Martin E. Garcia-Caceres C. Morselli E. Clegg D. J. Chowen J. A. Finan B.. (2013). Estrogen, astrocytes and the neuroendocrine control of metabolism. Rev. Endocr. Metab. Disord. 14, 331–338. doi: 10.1007/s11154-013-9263-7, PMID: 24009071 PMC3825572

[ref20] Galeotti N. Stefano G. B. Guarna M. Bianchi E. Ghelardini C. (2006). Signaling pathway of morphine induced acute thermal hyperalgesia in mice. Pain 123, 294–305. doi: 10.1016/j.pain.2006.03.008, PMID: 16650582

[ref21] Gao P. Ding X. W. Dong L. Luo P. Zhang G. H. Rong W. F. (2017). Expression of aromatase in the rostral ventromedial medulla and its role in the regulation of visceral pain. CNS Neurosci. Ther. 23, 980–989. doi: 10.1111/cns.12769, PMID: 29047208 PMC6492749

[ref22] Gao T. Dong L. Qian J. Ding X. Zheng Y. Wu M.. (2021a). G-protein-coupled estrogen receptor (GPER) in the rostral ventromedial medulla is essential for mobilizing descending inhibition of itch. J. Neurosci. 41, 7727–7741. doi: 10.1523/JNEUROSCI.2592-20.2021, PMID: 34349001 PMC8445050

[ref23] Gao T. Dong L. Qian J. Ding X. Zheng Y. Wu M.. (2021b). G-protein-coupled estrogen receptor (GPER) in the rostral ventromedial medulla is essential for mobilizing descending inhibition of itch. J. Neurosci 41, 7727–7741. 34349001 10.1523/JNEUROSCI.2592-20.2021 PMC8445050

[ref24] Guan Y. Guo W. Robbins M. T. Dubner R. Ren K. (2004). Changes in AMPA receptor phosphorylation in the rostral ventromedial medulla after inflammatory hyperalgesia in rats. Neurosci. Lett. 366, 201–205. doi: 10.1016/j.neulet.2004.05.051, PMID: 15276247

[ref25] Guan Y. Guo W. Zou S. P. Dubner R. Ren K. (2003). Inflammation-induced upregulation of AMPA receptor subunit expression in brain stem pain modulatory circuitry. Pain 104, 401–413. doi: 10.1016/s0304-3959(03)00048-4, PMID: 12855351

[ref26] Güldner F. H. Ingham C. A. (1980). Increase in postsynaptic density material in optic target neurons of the rat suprachiasmatic nucleus after bilateral enucleation. Neurosci. Lett. 17, 27–31. doi: 10.1016/0304-3940(80)90056-7, PMID: 6302580

[ref27] Hagino Y. Kariura Y. Manago Y. Amano T. Wang B. Sekiguchi M.. (2004). Heterogeneity and potentiation of AMPA type of glutamate receptors in rat cultured microglia. Glia 47, 68–77. doi: 10.1002/glia.20034, PMID: 15139014

[ref28] Handa S. Keith D. A. Abou-Ezzi J. Rosèn A. (2021). Neuropathic orofacial pain: characterization of different patient groups using the ICOP first edition, in a tertiary level Orofacial pain clinic. Oral Surg. Oral Med. Oral Pathol. Oral Radiol. 132, 653–661. doi: 10.1016/j.oooo.2021.07.021, PMID: 34518134

[ref29] Harris J. A. (1998). Using c-fos as a neural marker of pain. Brain Res. Bull. 45, 1–8. doi: 10.1016/S0361-9230(97)00277-3 9434195

[ref30] Héja L. Simon Á. Szabó Z. Kardos J. (2019). Feedback adaptation of synaptic excitability via Glu:Na+ symport driven astrocytic GABA and Gln release. Neuropharmacology 161:107629. doi: 10.1016/j.neuropharm.2019.05.006, PMID: 31103619

[ref31] Hucho T. B. Dina O. A. Kuhn J. Levine J. D. (2006). Estrogen controls PKCepsilon-dependent mechanical hyperalgesia through direct action on nociceptive neurons. Eur. J. Neurosci. 24, 527–534. doi: 10.1111/j.1460-9568.2006.04913.x, PMID: 16836642

[ref32] Iwata K. Sessle B. J. (2019). The evolution of neuroscience as a research field relevant to dentistry. J. Dent. Res. 98, 1407–1417. doi: 10.1177/0022034519875724, PMID: 31746682

[ref33] Jackson T. Thomas S. Stabile V. Han X. Shotwell M. McQueen K. (2015). Prevalence of chronic pain in low-income and middle-income countries: a systematic review and meta-analysis. Lancet 385:S10. doi: 10.1016/S0140-6736(15)60805-4, PMID: 26313056

[ref34] Kruyer A. Kalivas P. W. Scofield M. D. (2023). Astrocyte regulation of synaptic signaling in psychiatric disorders. Neuropsychopharmacology 48, 21–36. doi: 10.1038/s41386-022-01338-w, PMID: 35577914 PMC9700696

[ref35] Kuhn J. Dina O. A. Goswami C. Suckow V. Levine J. D. Hucho T. (2008). GPR30 estrogen receptor agonists induce mechanical hyperalgesia in the rat. Eur. J. Neurosci. 27, 1700–1709. doi: 10.1111/j.1460-9568.2008.06131.x, PMID: 18371086

[ref36] Kuo J. Hamid N. Bondar G. Prossnitz E. R. Micevych P. (2010). Membrane estrogen receptors stimulate intracellular calcium release and progesterone synthesis in hypothalamic astrocytes. J. Neurosci. 30, 12950–12957. doi: 10.1523/jneurosci.1158-10.2010, PMID: 20881113 PMC2957903

[ref37] Lau B. K. Vaughan C. W. (2014). Descending modulation of pain: the GABA disinhibition hypothesis of analgesia. Curr. Opin. Neurobiol. 29, 159–164. doi: 10.1016/j.conb.2014.07.010, PMID: 25064178

[ref38] Lee E. Sidoryk-Wêgrzynowicz M. Wang N. Webb A. Son D. S. Lee K.. (2012). GPR30 regulates glutamate transporter GLT-1 expression in rat primary astrocytes. J. Biol. Chem. 287, 26817–26828. doi: 10.1074/jbc.M112.341867, PMID: 22645130 PMC3411019

[ref39] Li C. Cai H. Meng Q. Feng Y. Guo H. Fang W.. (2016). IL-1beta mediating high mobility group box protein-1 expression in condylar chondrocyte during temporomandibular joint inflammation. J. Oral Pathol. Med. 45, 539–545. doi: 10.1111/jop.12401, PMID: 26671727

[ref40] Marvaldi L. Panayotis N. Alber S. Dagan S. Y. Okladnikov N. Koppel I.. (2020). Importin α3 regulates chronic pain pathways in peripheral sensory neurons. Science 369, 842–846. doi: 10.1126/science.aaz5875, PMID: 32792398

[ref41] Matute C. Domercq M. Fau-Sánchez-Gómez M. V. Sánchez-Gómez M. V. (2006). Glutamate-mediated glial injury: Mechanisms and clinical importance. Glia 53, 212–224. 16206168 10.1002/glia.20275

[ref42] McCarthy M. M. Amateau S. K. Mong J. A. (2002). Steroid modulation of astrocytes in the neonatal brain: implications for adult reproductive function. Biol. Reprod. 67, 691–698. doi: 10.1095/biolreprod.102.003251, PMID: 12193373

[ref43] Mo S.-Y. Xu X.-X. Bai S.-S. Liu Y. Fu K.-Y. Sessle B. J.. (2022). Neuronal activities in the rostral ventromedial medulla associated with experimental occlusal interference-induced orofacial hyperalgesia. J. Neurosci. 42, 5314–5329. doi: 10.1523/JNEUROSCI.0008-22.2022, PMID: 35667852 PMC9270923

[ref44] Murugan M. Ling Ea Fau-Kaur C. Kaur C. (2013). Glutamate receptors in microglia. CNS Neurol. Disord. Drug Targets 12, 773–784. 24047523 10.2174/18715273113126660174

[ref45] Olde B. Leeb-Lundberg L. M. (2009). GPR30/GPER1: searching for a role in estrogen physiology. Trends Endocrinol. Metab. 20, 409–416. doi: 10.1016/j.tem.2009.04.006, PMID: 19734054

[ref46] Ossipov M. H. Hong Sun T. Malan P. Jr. Lai J. Porreca F. (2000). Mediation of spinal nerve injury induced tactile allodynia by descending facilitatory pathways in the dorsolateral funiculus in rats. Neurosci. Lett. 290, 129–132. doi: 10.1016/s0304-3940(00)01338-0, PMID: 10936694

[ref47] Pedersen N. P. Vaughan C. W. Christie M. J. (2011). Opioid receptor modulation of GABAergic and serotonergic spinally projecting neurons of the rostral ventromedial medulla in mice. J. Neurophysiol. 106, 731–740. doi: 10.1152/jn.01062.2010, PMID: 21593395

[ref48] Radhakrishnan R. Sluka K. A. (2009). Increased glutamate and decreased glycine release in the rostral ventromedial medulla during induction of a pre-clinical model of chronic widespread muscle pain. Neurosci. Lett. 457, 141–145. 19429181 10.1016/j.neulet.2009.03.086 PMC2710144

[ref49] Ren K. (1999). An improved method for assessing mechanical allodynia in the rat. Physiol. Behav. 67, 711–716. doi: 10.1016/s0031-9384(99)00136-5 10604842

[ref50] Revankar C. M. Cimino D. F. Sklar L. A. Arterburn J. B. Prossnitz E. R. (2005). A transmembrane intracellular estrogen receptor mediates rapid cell signaling. Science 307, 1625–1630. doi: 10.1126/science.1106943, PMID: 15705806

[ref51] Slade G. D. Greenspan J. D. Fillingim R. B. Maixner W. Sharma S. Ohrbach R. (2020). Overlap of five chronic pain conditions: Temporomandibular disorders, headache, Back pain, irritable bowel syndrome, and fibromyalgia. J. Oral Facial Pain Headache 34, s15–s28. doi: 10.11607/ofph.2581, PMID: 32975538 PMC10073941

[ref52] Tao W. Chen Q. Zhou W. Wang Y. Wang L. Zhang Z. (2014). Persistent inflammation-induced up-regulation of brain-derived neurotrophic factor (BDNF) promotes synaptic delivery of alpha-amino-3-hydroxy-5-methyl-4-isoxazolepropionic acid receptor GluA1 subunits in descending pain modulatory circuits. J. Biol. Chem. 289, 22196–22204. doi: 10.1074/jbc.M114.580381, PMID: 24966334 PMC4139232

[ref53] Wang W. Zhong X. Li Y. Guo R. Du S. Wen L.. (2019). Rostral ventromedial medulla-mediated descending facilitation following P2X7 receptor activation is involved in the development of chronic post-operative pain. J. Neurochem. 149, 760–780. doi: 10.1111/jnc.14650, PMID: 30570747

[ref54] Wang Y. Pan Q. Tian R. Wen Q. Qin G. Zhang D.. (2021). Repeated oxytocin prevents central sensitization by regulating synaptic plasticity via oxytocin receptor in a chronic migraine mouse model. J. Headache Pain 22:84. doi: 10.1186/s10194-021-01299-3, PMID: 34315403 PMC8314458

[ref55] Wei F. Guo W. Zou S. Ren K. Dubner R. (2008). Supraspinal glial-neuronal interactions contribute to descending pain facilitation. J. Neurosci. 28, 10482–10495. doi: 10.1523/JNEUROSCI.3593-08.2008, PMID: 18923025 PMC2660868

[ref56] Wei L. Zhu Y. M. Zhang Y. X. Liang F. Jia H. Qu C. L.. (2016). Activation of alpha1 adrenoceptors in ventrolateral orbital cortex attenuates allodynia induced by spared nerve injury in rats. Neurochem. Int. 99, 85–93. doi: 10.1016/j.neuint.2016.06.006, PMID: 27296114

[ref57] Winkler C. W. Hermes S. M. Chavkin C. I. Drake C. T. Morrison S. F. Aicher S. A. (2006). Kappa opioid receptor (KOR) and GAD67 immunoreactivity are found in OFF and NEUTRAL cells in the rostral ventromedial medulla. J. Neurophysiol. 96, 3465–3473. doi: 10.1152/jn.00676.2006, PMID: 17005613

[ref58] Wu Y. W. Bi Y. P. Kou X. X. Xu W. Ma L. Q. Wang K. W.. (2010). 17-Beta-estradiol enhanced allodynia of inflammatory temporomandibular joint through upregulation of hippocampal TRPV1 in ovariectomized rats. J. Neurosci. 30, 8710–8719. doi: 10.1523/jneurosci.6323-09.2010, PMID: 20592193 PMC6632888

[ref59] Xu J. J. Gao P. Wu Y. Yin S. Q. Zhu L. Xu S. H.. (2021). G protein-coupled estrogen receptor in the rostral ventromedial medulla contributes to the chronification of postoperative pain. CNS Neurosci. Ther. 27, 1313–1326. doi: 10.1111/cns.13704, PMID: 34255932 PMC8504531

[ref60] Xu Z. Xie W. Feng Y. Wang Y. Li X. Liu J.. (2022). Positive interaction between GPER and beta-alanine in the dorsal root ganglion uncovers potential mechanisms: mediating continuous neuronal sensitization and neuroinflammation responses in neuropathic pain. J. Neuroinflammation 19:164. doi: 10.1186/s12974-022-02524-9, PMID: 35729568 PMC9215054

[ref61] Zeng J. Li S. Zhang C. Huang G. Yu C. (2018). The mechanism of Hyperalgesia and anxiety induced by Remifentanil: phosphorylation of GluR1 receptors in the anterior cingulate cortex. J. Mol. Neurosci. 65, 93–101. doi: 10.1007/s12031-018-1072-8, PMID: 29728964

